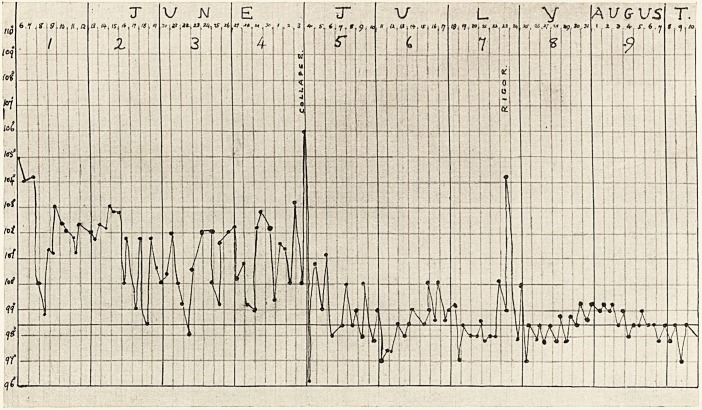# A Case of Typhoid Fever with Co-Existent Bacillus Coli Infection

**Published:** 1930

**Authors:** J. Innes Noble

**Affiliations:** Clifton


### A CASE OF TYPHOID FEVER WITH CO-EXISTENT BACILLUS COLI INFECTION.

BY

J. Innes Noble, M.B., Ch.B., F.R.C.S.
(*Clifton*).

---

This case seems to merit detailed description by reason of (1) obscurity of diagnosis, (2) atypical course, (3) difficulty in treating the coincident infections, (4) "heroics" in treatment demanded by the condition, (5) complete recovery of the patient.

The patient was a woman, aged 41, unmarried, of the habitually constipated, visceroptotic type. Two pounds in weight at birth, she had had infantile bowel disorders, and from puberty onwards had suffered from intermittent attacks of what was probably a *B. coli* infection, characterized by general malaise, severe headache and elevation of temperature. She constantly took cathartics.

*Source of Infection.*—Symptoms of departure from normal health developed ten days after the patient had returned to this country from South Africa, where she had been for six months. A case of "fever" had been put ashore at Gibraltar which might have been one of typhoid. On the other hand, infection may have occurred from contaminated food taken on board at Madeira.

*History of Incubation and Onset.*—Ten days after return the patient experienced severe frontal headache and recognized that there was a slight rise of temperature. Lassitude was pronounced, and an unusual shrinking from effort noticed. She pursued her usual mode of life, with occasional days in bed, for ten days. Then followed three consecutive days' confinement to bed with no remission of symptoms except a return to normal temperature on the third day of recumbency. She again resumed active life with attendance at social functions for another week. During this week headache and lassitude persisted. The patient finally took to bed twenty-three days after the first sensation of feeling unwell, and was in bed when I saw her for the first time, eight days later. The following *symptoms* were noted :—

A. *Subjective.*—(1) Headache, maximum in intensity in the frontal region and not relieved by salicylates. (2) Pain of a boring character in the lower lumbar region and in the front of both thighs. (3) Epistaxis which had occurred twice in the preceding week, but had not been considered noteworthy because nose-bleeding had been a frequent occurrence in the past. (4) General malaise, with inability to concentrate in any way. (5) Constipation. There had been only one bowel evacuation in the week.

There was no history of cough, dysuria, vomiting or shivering.

B. *Objective.*—The patient lay partially propped up in bed. She looked dull and lethargic. There was a malar flush. Questions were answered hesitatingly, with slow reaction. The attention was arrested by her apathy and fixation of gaze. The forehead was wrinkled and the lips dry and parted. The skin was dry. The conjunctivæ were earthy in colour and slightly icteroid. The tongue was dry, rather shrivelled, red-raw at the tip and in the centre, but coated on the sides. The temperature was 105° F.

The abdomen was distended uniformly. It moved equally on respiration, and there was no visible lump. There was general tenderness on palpation and a peculiar doughy feel to the hand, especially over the cæcum and over the pelvic colon. Pain on pressure was complained of over the sigmoid anteriorly and in the renal areas posteriorly. There was no rigidity and the liver dullness was normal. The percussion note was uniformly tympanitic. The rectum was empty and felt very hot to the finger. The spleen was not palpable.

There was a defective percussion note at the right base. The breath sounds were normal throughout. There were no adventitious sounds. The respirations were 18 per minute.

The heart was normal in size. The impulse was intense. There were no adventitious sounds. The pulse was 120 per minute, regular, full and bounding. The systolic blood-pressure was 135 mms. Hg.

The kidneys were not palpable. Pain already referred to was complained of on pressure in the renal areas. The urine was acid, 1028. Albumin and pus were present. A catheter specimen was taken for bacteriological examination.

All reflexes were normal. The pupils were equally contracted. Reaction time to questions was retarded. There were no other abnormalities found.

20 ccs. of blood were taken for examination. From this a positive Widal reaction was obtained, at an agglutination of 1–250 ; and *B. typhosus* was grown in culture.

The urine showed quantities of pus cells, and a pure culture of *B. coli* was grown.

A *diagnosis* of typhoid fever with complicating *B. coli* infection was made from the bacteriological findings. The spleen was not palpable at this stage of the disease, but was felt four days later. The subsequent development of *B. coli* septicæmia was proved by the abscess which occurred in the right biceps, the pus from which was apparently of the type associated with *B. coli*.

*Course.*—The general condition of the patient gradually became worse, until she lapsed into a state of stupor with low delirium and soon typified the typhoid state. It is impossible to give a detailed account of the course or the vagaries of the maladies, as they were protean. The following complications and unusual symptoms have been singled out as being most noteworthy :—

Constipation was absolute throughout. Until the fourth week the urine was scanty, and was not passed voluntarily until the fifth week. Muscle and superficial abscesses occurred in the right arm, the abscess in muscle being in the right biceps. Hypostatic congestion at both bases was noted, and persisted from the second until the fourth week. Œdema of the glottis occurred on the twenty-eighth day and was transient. Skin rashes of three kinds were seen, viz. (*a*) patchy erythema occurring in the second week and covering the entire body, apparently due to repeated turpentine enemata; (*b*) purpuric spots on both arms and shoulders, which were observed in the third week; (*c*) rose spots, first seen on the eighth day, and profuse on the abdomen, back, chest and arms. Conjunctivitis was noticed during the fourth week. It yielded to instillation of 1 per cent. mercurochrome.

During convalescence there was a curious delusion. This was the patient's belief that she had acquired a diamond mine, and she was dissuaded only with difficulty from signing cheques for large amounts to charitable institutions. This delusion persisted for a month.

Sudden collapse occurred on the eighteenth day, and was associated with increased frequency of the pulse, pallor and coldness of the skin, and sighing respirations. There were no signs of perforation. From the findings when bowel evacuation had been achieved, it is probable that a small hemorrhage occurred.

*Treatment.*—Owing to the confined state of the bowel the usual milk diet was impossible. Plain water and beef tea were the chief fluids given, and these were augmented by albumen water, glucose in water, barley water, and orange juice. At least six pints of plain water were given daily. It was feared that the bouillon would be harmful to the kidneys in the infected condition, but it was continued. Solids were not given until the sixth week, and even then all milky foods were refused by the patient, who had always been unable to take milk in any way.

The following methods of treating the various symptoms were employed :—

*Constipation.*—Enemata—soap and water, olive oil and

JUNE
JULY
AUGUST
COLLAPSE
RIGOR
1 2 3 4 5 6 7 8 9
110°
109°
108°
107°
106°
105°
104°
103°
102°
101°
100°
99°
98°
97°
96°

turpentine—were quite ineffectual, but were repeated frequently. Calomel was exhibited, primarily in $\frac{1}{2}$ grain doses until 5 grains had been given. This was followed by an enema, but no result was obtained. It was repeated in $\frac{1}{10}$ grain doses until 3 grains had been taken. Again there was no result. Paraffin was given as petrolagar until 6 ounces had been taken. No result was obtained and no vomiting occurred. From the fourth until the thirtieth day, after the previous methods had failed, recourse was had to bowel lavage. A tube 8 in. in length was inserted into the rectum, and two pints of warm soap and water were run into the bowel from a height. Each washing brought away soft motion, mucus and pus, and never failed to relieve abdominal distension. This lavage was carried out twice daily for seventeen consecutive days, and saved the patient's life.

Following a rigor and considered preponderance of the *B. coli* infection pituitrin was given on the thirtieth day. $\frac{1}{2}$ cc. was given at six-hourly intervals until $1\frac{1}{2}$ cc. had been given. There was no result. 1 cc. was administered after a further interval of six hours, and this was followed by two enormous motions containing quantities of pus, altered blood and mucus.

The bowel was kept free after this by "cascara evacuant."

*Suppression of Urine.*—The urine was scanty in amount and no voluntary evacuation of the bladder was possible, as described. Catheterization was necessary twice daily until the twenty-eighth day. Mercurochrome 1 per cent. was put into the bladder daily, six ounces being allowed to remain in for two hours.

After the flow of urine had again been established, pyridium was exhibited for three weeks. Two tablets were given twice daily. Prior to this hexamine in 10 grain doses had been given, followed by large doses of acid sodium phosphate. This had no effect on the *B. coli* infection. It was found later in the course of the disease that large doses of potassium citrate and sodium bicarbonate had the greatest beneficial effect on the urine.

*Muscle Abscess.*—Free incision was made and a rubber dam drain inserted.

*Sudden Collapse.*—Subcutaneous salines with glucose were given continuously by means of a Lane's bag. Strychnine gr. $\frac{1}{30}$ was repeated until twitching occurred. This drug was exhibited throughout the critical stages of the malady, and with coramine ("Ciba") proved the most valuable stimulant.

*Headache.*—Phenazonum, gr. 3, three-hourly, proved efficacious in the relief of this symptom, which was distressing in the first week.

*Distension.*—This was relieved by bowel lavage as previously described, by administration of oleum cinnamomi, ♏.3 in capsules, four hourly and by the flatus tube.

Massage was administered to the limbs and back as soon as convalescence was established.

The patient was allowed to sit out of bed before the night temperature was steadily normal.

### SUMMARY.

The writer has submitted these notes because of their possible use to the general practitioner, whose therapeutic aids must necessarily come from standard works and his own or others' experience. The dictum on the treatment of constipation in the most recent work is: "It is well to give every second day an ordinary enema. The addition of turpentine is advisable if there is meteorism."

At least twenty enemata were given in this case without result. Without free bowel action the treatment of *B. coli* infection is impossible, and here we were dealing with paralysed bowel, intensely delicate from typhoid ulceration. The very fact of being compelled to submit a typhoid intestine to the stress of pituitrin seems to excuse this record. One of the most curious things in the history of this case is the fact that throughout vomiting occurred only once, after the first administration of calomel.

The patient is now able to pursue a moderately active life.

The sincere thanks of the writer are due to Professor Nixon and to Dr. Carey Coombs for their help and interest in the case.

## Figures and Tables

**Figure f1:**